# Poly[aqua­(μ_5_-2-oxido-4-sulfonato­benzoato)lanthanum(III)]

**DOI:** 10.1107/S1600536809007879

**Published:** 2009-03-11

**Authors:** Cheng-Feng Zhu, Xing Li, Enhong Sheng

**Affiliations:** a College of Chemistry and Materials Science, Anhui Normal University, Wuhu, Anhui 241000, People’s Republic of China; b Faculty of Materials Science and Chemical Engineering, Ningbo University, Ningbo, Zhengjiang 315211, People’s Republic of China

## Abstract

The title compound, [La(C_7_H_3_O_6_S)(H_2_O)]_*n*_, forms a three-dimensional framework in which the asymmetric unit contains one La^III^ atom, one 5-sulfosalicylate (2-oxido-4-sulfonatobenzoate) ligand and one coordinated water mol­ecule. The La^III^ atom is coordinated by nine O atoms from three carboxyl­ate, three sulfonate and two hydroxyl groups, and one water mol­ecule, forming a distorted trigonal-prismatic square-face tricapped geometry.

## Related literature

For the use of rigid carboxyl­ate ligands in the design and synthesis of a variety of structures, see: Cao *et al.* (2002 ▶); Li *et al.* (2004 ▶, 2005 ▶). For the structure of the isotypic Nd compound, see: Wang *et al.* (2004 ▶).
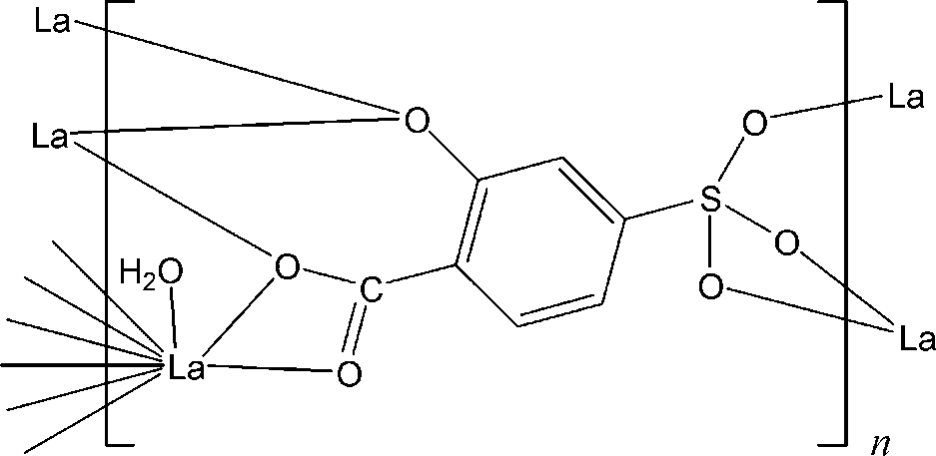


## Experimental

### 

#### Crystal data

[La(C_7_H_3_O_6_S)(H_2_O)]
*M* *_r_* = 372.08
Triclinic, 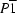

*a* = 6.2297 (7) Å
*b* = 8.2390 (8) Å
*c* = 9.9157 (9) Å
α = 111.587 (2)°
β = 94.325 (2)°
γ = 93.785 (2)°
*V* = 469.47 (8) Å^3^
*Z* = 2
Mo *K*α radiation
μ = 4.79 mm^−1^
*T* = 293 K
0.24 × 0.16 × 0.12 mm

#### Data collection

Siemens SMART CCD area-detector diffractometer
Absorption correction: multi-scan (**SADABS**; Sheldrick, 1996 ▶) *T*_min_ = 0.415, *T*_max_ = 0.563
2457 measured reflections
1633 independent reflections
1527 reflections with *I* > 2σ(*I*)
*R*_int_ = 0.029

#### Refinement

*R*[*F* ^2^ > 2σ(*F* ^2^)] = 0.035
*wR*(*F* ^2^) = 0.090
*S* = 1.07
1633 reflections
145 parameters
H-atom parameters constrained
Δρ_max_ = 0.79 e Å^−3^
Δρ_min_ = −1.66 e Å^−3^

### 

Data collection: *SMART* (Siemens, 1996 ▶); cell refinement: *SAINT* (Siemens, 1994 ▶); data reduction: *SAINT*; program(s) used to solve structure: *SHELXS97* (Sheldrick, 2008 ▶); program(s) used to refine structure: *SHELXL97* (Sheldrick, 2008 ▶); molecular graphics: *SHELXTL* (Sheldrick, 2008 ▶); software used to prepare material for publication: *SHELXTL*.

## Supplementary Material

Crystal structure: contains datablocks I, global. DOI: 10.1107/S1600536809007879/is2373sup1.cif

Structure factors: contains datablocks I. DOI: 10.1107/S1600536809007879/is2373Isup2.hkl

Additional supplementary materials:  crystallographic information; 3D view; checkCIF report

## Figures and Tables

**Table 1:** Selected bond lengths (Å)

La1—O1^i^	2.676 (5)
La1—O2^ii^	2.449 (5)
La1—O2^i^	2.561 (5)
La1—O3^iii^	2.478 (4)
La1—O3^ii^	2.499 (4)
La1—O4	2.573 (5)
La1—O5	2.970 (7)
La1—O6^iv^	2.548 (5)
La1—O7	2.501 (5)

Symmetry codes: (i) 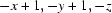; (ii) 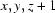; (iii) 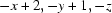; (iv) 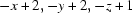.

**Fig. 1.**
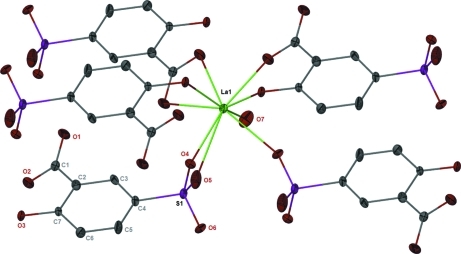
A view of the lanthanum ion coordination, Displacement ellipsoids are drawn at the 50% probability level.

**Fig. 2.**
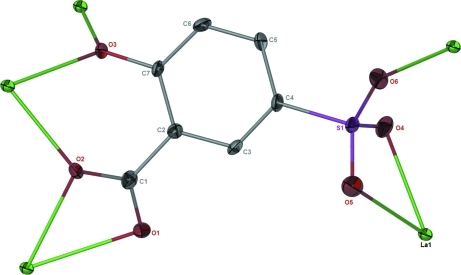
The sulfosalicylate ligands linking fashion.

## supplementary crystallographic information

### Comment

The carboxylate groups have a strong ability to bond various metal ions and afford abundant coordination modes, thus rigid carboxylate ligands have been widely used for the design and synthesis of a great variety of structures (Cao *et al.*, 2002; Li *et al.*, 2004). Introduction of a sulfonate group into rigid carboxylate ligands may result in the formation of unexpected frameworks as the sulfonic group has a different shape and properties in terms of its coordination ability compared to the carboxylate group (Li *et al.*, 2005).

Studies on the coordination chemistry of mixed carboxylate-sulfonic ligands are not very common. By employing 5-sulfoisophthalic acid as an organic ligand, we have successfully prepared one new metal-organic polymer [La(C_7_H_3_O_6_S)(H_2_O)]_n_ with three dimensional framework by the bridging carboxylate and the sulfonate groups. Single X-ray diffraction analysis reveals that the title complex is isomorphous to the La-compound reported previously (Wang *et al.*, 2004). As Fig. 1 shown, the lanthanum(III) atom is surrounded by nine oxygen atoms, of which three come from carboxylate groups, three from sulfonates, two from hydroxyl groups and one from the coordinated water. The sulfosalicylate ligands serve as µ_5_-bridge linking five La(III) ions (Fig. 2), resulting in a three dimensional framework.

### Experimental

A mixture of 5-sulfosalicylic acid (0.60 mmol, 0.15 g), La_2_O_3_ (0.2 mmol, 0.065 g) and H_2_O (15 ml) was sealed in a 25 ml stainless steel reactor with Teflon linear and heated 433 K for 72 h. After cooling to room temperature, colorless crystals were isolated by filtering (yield 35%).

### Refinement

Aromatic hydrogen atoms were assigned to calculated positions and allowed to ride on their respective parent C atoms with isotropic thermal displacement parameters. For the hydrogen atoms bonded to coordination water oxygen atom, The H7A was positioned geometrically and H7B was located in a difference map and constrained with O—H = 0.82 Å. The deepest residual electron density peak is located at 0.87 Å from atom La1.

### Crystal data

**Table:**

[La(C_7_H_3_O_6_S)(H_2_O)]	*Z* = 2
*M**_r_* = 372.08	*F*(000) = 352
Triclinic, *P*1	*D*_x_ = 2.632 Mg m^−^^3^
Hall symbol: -P 1	Mo *K*α radiation, λ = 0.71073 Å
*a* = 6.2297 (7) Å	Cell parameters from 2088 reflections
*b* = 8.2390 (8) Å	θ = 2.2–25.1°
*c* = 9.9157 (9) Å	µ = 4.79 mm^−^^1^
α = 111.587 (2)°	*T* = 293 K
β = 94.325 (2)°	Block, colorless
γ = 93.785 (2)°	0.24 × 0.16 × 0.12 mm
*V* = 469.47 (8) Å^3^	

### Data collection

**Table:**

Siemens SMART CCD area-detector diffractometer	1633 independent reflections
Radiation source: fine-focus sealed tube	1527 reflections with *I* > 2σ(*I*)
graphite	*R*_int_ = 0.029
ω scans	θ_max_ = 25.1°, θ_min_ = 2.2°
Absorption correction: multi-scan (*SADABS*; Sheldrick, 1996)	*h* = −4→7
*T*_min_ = 0.415, *T*_max_ = 0.563	*k* = −9→9
2457 measured reflections	*l* = −11→11

### Refinement

**Table:**

Refinement on *F*^2^	Primary atom site location: structure-invariant direct methods
Least-squares matrix: full	Secondary atom site location: difference Fourier map
*R*[*F*^2^ > 2σ(*F*^2^)] = 0.035	Hydrogen site location: inferred from neighbouring sites
*wR*(*F*^2^) = 0.090	H-atom parameters constrained
*S* = 1.07	*w* = 1/[σ^2^(*F*_o_^2^) + (0.0456*P*)^2^ + 4.598*P*] where *P* = (*F*_o_^2^ + 2*F*_c_^2^)/3
1633 reflections	(Δ/σ)_max_ < 0.001
145 parameters	Δρ_max_ = 0.79 e Å^−^^3^
0 restraints	Δρ_min_ = −1.66 e Å^−^^3^

### Special details

**Table:**

Geometry. All e.s.d.'s (except the e.s.d. in the dihedral angle between two l.s. planes) are estimated using the full covariance matrix. The cell e.s.d.'s are taken into account individually in the estimation of e.s.d.'s in distances, angles and torsion angles; correlations between e.s.d.'s in cell parameters are only used when they are defined by crystal symmetry. An approximate (isotropic) treatment of cell e.s.d.'s is used for estimating e.s.d.'s involving l.s. planes.
Refinement. Refinement of *F*^2^ against ALL reflections. The weighted *R*-factor *wR* and goodness of fit *S* are based on *F*^2^, conventional *R*-factors *R* are based on *F*, with *F* set to zero for negative *F*^2^. The threshold expression of *F*^2^ > σ(*F*^2^) is used only for calculating *R*-factors(gt) *etc*. and is not relevant to the choice of reflections for refinement. *R*-factors based on *F*^2^ are statistically about twice as large as those based on *F*, and *R*- factors based on ALL data will be even larger.

### Fractional atomic coordinates and isotropic or equivalent isotropic displacement parameters (Å^2^)

**Table:**

	*x*	*y*	*z*	*U*_iso_*/*U*_eq_	
La1	0.76441 (5)	0.64763 (5)	0.46004 (4)	0.01280 (16)	
S1	0.9837 (3)	0.8865 (2)	0.28204 (17)	0.0167 (4)	
O1	0.4823 (8)	0.4571 (7)	−0.2043 (5)	0.0258 (12)	
O2	0.5737 (8)	0.5722 (7)	−0.3605 (5)	0.0215 (11)	
O3	1.0224 (7)	0.6040 (6)	−0.3536 (5)	0.0139 (9)	
O4	1.0612 (9)	0.7528 (6)	0.3353 (5)	0.0229 (11)	
O5	0.7603 (9)	0.9023 (8)	0.3122 (6)	0.0360 (14)	
O6	1.1278 (9)	1.0487 (6)	0.3439 (5)	0.0237 (11)	
O7	0.4427 (9)	0.8190 (7)	0.5052 (7)	0.0332 (13)	
H7A	0.3472	0.7722	0.4375	0.040*	
H7B	0.4185	0.9029	0.5766	0.040*	
C1	0.6163 (12)	0.5488 (9)	−0.2412 (7)	0.0181 (14)	
C2	0.8185 (11)	0.6385 (9)	−0.1475 (7)	0.0150 (13)	
C3	0.8153 (11)	0.7035 (9)	0.0033 (7)	0.0153 (13)	
H3A	0.6938	0.6777	0.0437	0.018*	
C4	0.9937 (11)	0.8069 (9)	0.0930 (7)	0.0168 (14)	
C5	1.1779 (12)	0.8448 (10)	0.0340 (7)	0.0210 (15)	
H5A	1.2964	0.9148	0.0953	0.025*	
C6	1.1846 (11)	0.7790 (9)	−0.1142 (8)	0.0192 (14)	
H6A	1.3085	0.8041	−0.1526	0.023*	
C7	1.0069 (11)	0.6740 (8)	−0.2089 (7)	0.0141 (13)	

### Atomic displacement parameters (Å^2^)

**Table:**

	*U*^11^	*U*^22^	*U*^33^	*U*^12^	*U*^13^	*U*^23^
La1	0.0098 (2)	0.0143 (2)	0.0121 (2)	−0.00092 (15)	0.00131 (14)	0.00277 (16)
S1	0.0185 (8)	0.0174 (8)	0.0098 (8)	−0.0033 (7)	0.0027 (6)	0.0005 (6)
O1	0.023 (3)	0.034 (3)	0.019 (3)	−0.016 (2)	−0.001 (2)	0.012 (2)
O2	0.017 (2)	0.031 (3)	0.016 (3)	−0.009 (2)	−0.005 (2)	0.012 (2)
O3	0.015 (2)	0.015 (2)	0.009 (2)	0.0004 (18)	0.0007 (18)	0.0012 (18)
O4	0.034 (3)	0.019 (2)	0.017 (3)	−0.002 (2)	0.005 (2)	0.009 (2)
O5	0.025 (3)	0.047 (4)	0.022 (3)	0.001 (3)	0.008 (2)	−0.004 (3)
O6	0.031 (3)	0.014 (2)	0.020 (3)	−0.010 (2)	−0.001 (2)	0.001 (2)
O7	0.023 (3)	0.030 (3)	0.037 (3)	0.010 (2)	0.003 (2)	0.000 (3)
C1	0.024 (4)	0.014 (3)	0.014 (3)	0.000 (3)	0.004 (3)	0.002 (3)
C2	0.014 (3)	0.018 (3)	0.014 (3)	0.004 (3)	0.003 (3)	0.005 (3)
C3	0.017 (3)	0.019 (3)	0.012 (3)	0.003 (3)	0.007 (3)	0.007 (3)
C4	0.022 (3)	0.018 (3)	0.008 (3)	−0.002 (3)	0.001 (3)	0.003 (3)
C5	0.019 (3)	0.028 (4)	0.010 (3)	−0.003 (3)	−0.001 (3)	0.001 (3)
C6	0.017 (3)	0.020 (3)	0.017 (3)	−0.003 (3)	0.008 (3)	0.002 (3)
C7	0.019 (3)	0.014 (3)	0.008 (3)	−0.001 (3)	0.002 (3)	0.003 (3)

### Geometric parameters (Å, °)

**Table:**

La1—O1^i^	2.676 (5)	O2—C1	1.279 (9)
La1—O2^ii^	2.449 (5)	O3—C7	1.349 (8)
La1—O2^i^	2.561 (5)	O7—H7A	0.8200
La1—O3^iii^	2.478 (4)	O7—H7B	0.8200
La1—O3^ii^	2.499 (4)	C1—C2	1.480 (10)
La1—O4	2.573 (5)	C2—C3	1.393 (9)
La1—O5	2.970 (7)	C2—C7	1.424 (9)
La1—O6^iv^	2.548 (5)	C3—C4	1.385 (10)
La1—O7	2.501 (5)	C3—H3A	0.9300
S1—O4	1.478 (5)	C4—C5	1.395 (10)
S1—O5	1.450 (6)	C5—C6	1.372 (10)
S1—O6	1.457 (5)	C5—H5A	0.9300
S1—C4	1.751 (7)	C6—C7	1.405 (10)
O1—C1	1.251 (9)	C6—H6A	0.9300
			
O2^ii^—La1—O3^iii^	103.65 (16)	O6—S1—O4	110.7 (3)
O2^ii^—La1—O3^ii^	68.47 (15)	O5—S1—C4	109.1 (3)
O3^iii^—La1—O3^ii^	67.24 (16)	O6—S1—C4	107.0 (3)
O2^ii^—La1—O7	72.75 (19)	O4—S1—C4	107.5 (3)
O3^iii^—La1—O7	158.70 (17)	C1—O1—La1^i^	93.1 (4)
O3^ii^—La1—O7	127.17 (17)	C1—O2—La1^v^	139.2 (4)
O2^ii^—La1—O6^iv^	87.98 (17)	C1—O2—La1^i^	97.8 (4)
O3^iii^—La1—O6^iv^	131.23 (16)	La1^v^—O2—La1^i^	116.49 (18)
O3^ii^—La1—O6^iv^	74.06 (16)	C7—O3—La1^iii^	121.3 (4)
O7—La1—O6^iv^	70.05 (18)	C7—O3—La1^v^	123.5 (4)
O2^ii^—La1—O2^i^	63.51 (18)	La1^iii^—O3—La1^v^	112.76 (16)
O3^iii^—La1—O2^i^	86.88 (16)	S1—O4—La1	110.1 (3)
O3^ii^—La1—O2^i^	116.96 (15)	S1—O5—La1	93.3 (3)
O7—La1—O2^i^	72.64 (18)	S1—O6—La1^iv^	151.0 (3)
O6^iv^—La1—O2^i^	138.33 (17)	La1—O7—H7A	109.5
O2^ii^—La1—O4	162.22 (17)	La1—O7—H7B	130.5
O3^iii^—La1—O4	73.55 (15)	H7A—O7—H7B	119.7
O3^ii^—La1—O4	94.76 (16)	O1—C1—O2	119.2 (6)
O7—La1—O4	116.21 (18)	O1—C1—C2	122.1 (6)
O6^iv^—La1—O4	81.48 (16)	O2—C1—C2	118.6 (6)
O2^i^—La1—O4	132.71 (16)	C3—C2—C7	120.0 (6)
O2^ii^—La1—O1^i^	110.68 (15)	C3—C2—C1	118.6 (6)
O3^iii^—La1—O1^i^	88.69 (16)	C7—C2—C1	121.1 (6)
O3^ii^—La1—O1^i^	154.12 (16)	C4—C3—C2	119.7 (6)
O7—La1—O1^i^	73.65 (18)	C4—C3—H3A	120.1
O6^iv^—La1—O1^i^	131.55 (17)	C2—C3—H3A	120.1
O2^i^—La1—O1^i^	49.19 (15)	C3—C4—C5	120.7 (6)
O4—La1—O1^i^	86.95 (15)	C3—C4—S1	118.5 (5)
O2^ii^—La1—O5	139.80 (16)	C5—C4—S1	120.8 (5)
O3^iii^—La1—O5	115.64 (15)	C6—C5—C4	120.1 (6)
O3^ii^—La1—O5	134.13 (15)	C6—C5—H5A	120.0
O7—La1—O5	67.57 (18)	C4—C5—H5A	120.0
O6^iv^—La1—O5	72.93 (16)	C5—C6—C7	120.9 (6)
O2^i^—La1—O5	108.88 (15)	C5—C6—H6A	119.5
O4—La1—O5	49.58 (16)	C7—C6—H6A	119.5
O1^i^—La1—O5	64.08 (16)	O3—C7—C6	119.5 (6)
O5—S1—O6	115.5 (3)	O3—C7—C2	122.0 (6)
O5—S1—O4	106.9 (3)	C6—C7—C2	118.4 (6)

Symmetry codes: (i) −*x*+1, −*y*+1, −*z*; (ii) *x*, *y*, *z*+1; (iii) −*x*+2, −*y*+1, −*z*; (iv) −*x*+2, −*y*+2, −*z*+1; (v) *x*, *y*, *z*−1.
